# Supplementary material for: Savoring the present: The reciprocal influence between positive emotions and positive emotion regulation in everyday life
Source: PLoS One. 2021 May 11;16(5):e0251561. doi: 10.1371/journal.pone.0251561 (PMC8112694; doi:10.1371/journal.pone.0251561)
Supplement: Attachment — Submitted filename: Response to reviewers.docx [file pone.0251561.s001.docx]

**Reviewer #1**

*My biggest concern with the current study is that the factorial and construct validity of the use of ad-hoc single items to assess the implementation of positive strategies. Although in the discussion, the authors state that “the use of ad-hoc single items to assess a multifaceted construct like emotion regulation might have not fully captured the complexity of this process”, the explanation that “no validated questionnaire assessing state positive regulation has been so far developed” is not sufficient.*

Thank you for raising this point. As mentioned in the discussion, the use of single items to assess positive strategies might represent a criticism of the study. However, to the best of our knowledge, there aren’t validated items assessing state (e.g., momentary) positive regulation. The available questionnaires usually assess positive emotion regulation in a more general way (see for example the *Response Style to Positive Affect Scale*, which assesses emotion-focused or self-focused savoring; the *Savoring Belief Inventory*, which only explores savoring in terms of anticipating, experiencing or recalling positive states; or the *Emotion Regulation Profile-Revised*, which consists of narratives rather than items). Additionally, most of the available emotion regulation questionnaires relies on the idea that emotion regulation is a trait of a person, thus assessing emotion regulation as a cross-situational tendency. Only a few questionnaires have been developed to assess state emotion regulation (i.e., the momentary adoption of strategies in relation to a specific event, which would fit better with the use of EMA), but only in relation to negative emotions (see for example the *Brief State Rumination Inventory*, Marchetti et al., 2018; or the *State Difficulties in Emotion Regulation Scale*, Lavender et al., 2017). Finally, there is evidence showing that longer EMA questionnaires usually imply higher perceived burden and lower compliance to the experimental protocol, which further support our choice to use single items. Thus, integrating a full questionnaire of positive regulation in the EMA would have been counterproductive.

Broadly talking, the use of single items isn’t just a limitation of our study, but a very important issue of the EMA literature. Indeed, almost all EMA studies rely on the use of ad-hoc items, thus raising important issues regarding their construct validity. We acknowledge this criticism, and we recognise that there is the need to further expand this research field by creating validated measures to be used in momentary assessments. These efforts have been already made in other research fields. An example is the *Distress Thermometer*, a single measure which has been developed to assess “unpleasant experience of a mental, physical, social, or spiritual nature”. Similarly, validated items to assess emotion regulation should be created in the near future.

As the reviewer will see, we have tried to incorporate the aforementioned considerations in the manuscript and to better justify the use of single items (from line 484).

*Second, the classification of positive strategies may appear arbitrary. For example, the authors categorised two strategies, broadening and count blessing, into the “cognitive change” group. The item that used to assess “broadening” strategy is that “I’m thinking about all the good things that I have and that are happening in my life as well. ” and the item that used to assess count blessing is that “I’m thinking about how lucky I am to live this moment and feel so good. However, within the process model of emotion regulation (Gross, 1998, 2015), the core element of cognitive change is modifying the meaning of the situation to alter an emotional response. Theoretically, they are different strategies.*

This is a very interesting point, thank you for the comment. Our study was based on Gross’ model of emotion regulation (2015), which was originally thought for the regulation of negative affective states. Recently, Quoidbach et al. (Quoidbach, Mikolajczak and Gross, 2015) applied Gross’ model to explain and understand the regulation of positive emotions. Accordingly, our study was inspired by Quoidbach et al.’s theory.

In their model, Quoidbach et al. suggest counting blessings (“*Are you realizing how lucky you are or are you taking the situation for granted?*”) as an example of cognitive change in response to positive emotions, which is described as the attempt to “*change how one appraises the situation in order to alter its emotional significance”* by “*appraising the situation as a special moment*” or “*by increasing how valuable positive events appear*”. We therefore decided to include count blessing as a cognitive change strategy. Furthermore, we included broadening as a further example of cognitive change because it does reflect a way of appraising a positive situation and generalizing it to one’s positive things in life, which is consistent with the aforementioned definition.

Regarding the second category, Quoidbach et al. define attentional deployment as the attempt to influence positive emotions by specifically directing the attention within a situation (“*What do you purposefully pay attention to during your date night*?”). Accordingly, we decided to include mindfulness (I’m trying to be focused on the present and to concentrate on how good I feel) and stimulus control (I’m trying to avoid all negative thoughts and stressors in order to focus and take the most of my positive emotions) as two strategies that reflects the attempt to modify one’s attentional focus in order to take the most out of a positive emotion.

Finally, Quoidbach et al. describe response modulation as the attempt to “*influence physiological, experiential, or behavioral responding as directly as possible*” and suggest social sharing and emotional expression as two examples of this category (“*Are you laughing and sharing your positive feelings with your partner or, conversely, hiding your emotions?”).* We therefore decided to include both strategies to represent this category.

We do completely agree with the reviewer that clearer definitions of the categories were missing. We have now substantially modified the manuscript in order to clarify these points Moreover, and consistent with reviewer#2’s suggestion to explain why we expected those strategies to increase positive emotions, we have also tried to further justify the selection of the six specific strategies included in the study (from line 192; from line 198).

*Third, as a measure of the momentary intensity of positive emotions, participants were asked to rate 7 adjectives on a 1-5 Likert, including “happiness, amusement, hope, serenity, excitement, pride, gratitude”. However, the reasons for choosing these adjectives are not clarified.*

Thank you for raising this issue. We have now included more details regarding the selection of the seven positive emotions. More specifically, a previous study found that the regulation of positive emotions is likely to affect both active (e.g. happiness) and deactive (e.g., calmness) positive affect (Nezleck et al., 2008). Accordingly, we selected the seven emotions in order to include both low- (hope, serenity, gratitude) and high-arousal (happiness, amusement, excitement, pride) positive emotions. This information has been now included in the manuscript (from line 184).

*Fourth, the authors mention in their discussion that “upregulating PE may therefore serve as a mechanism to repair mood and reduce the experience of unpleasant affective states”. This conclusion is arbitrary due to it may be based on the assumption that PA and NA are on two opposite ends of a bipolar scale (i.e., People can be experiencing one type of affect to a certain degree but not the other at the same time). Indeed, there is a growing body of evidence that positive and negative affect can co-occur simultaneously (e.g., Berrios, Totterdell, & Kellett, 2015; Larsen & McGraw, 2011).*

We completely understand the reviewer’s point. As correctly mentioned by the reviewer, and consistent with an extensive literature, experiencing low levels of positive emotions does not imply experiencing high levels of negative affect. Indeed, the fact that we only assessed momentary PE could be considered a limitation of the study, as it does not allow to fully comprehend the reciprocal influence between positive emotion regulation and momentary affect (both positive and negative). We therefore decided to move this statement to the limitations of the study and to discuss it in more details. More specifically, we state that, despite the results might support the mood-repair theory, such conclusion can’t be confirmed because of the lack of information regarding momentary negative affect (from line 470).

*Fifth, the authors mention in the introduction “people who frequently adopt savouring strategies, for instance, show higher happiness, greater positive affect and enhanced emotional well-beings ” (Page 4, Paraphrase 2). However, the meaning of “savouring strategies” did not clearly defined at any other point in the manuscript.*

Thank you very much for pointing out this issue. We have now included a short definition of “savoring strategies” in the introduction (line 96).

**Reviewer #2**

*1. The introduction mentions multiple ways people can regulate emotions (e.g., Gross’s process model and Bryant’s conceptualization of savoring past, present, future), but these aren’t well integrated or applied to the current study. It is recommended that the authors are more explicit about how frameworks relate and inform the current study.*

Thank you for the suggestion. The introduction has been significantly revised in order to conceptualize in a clearer way the different frameworks related to the regulation of positive emotion regulation. More specifically, we have now underlined that, despite emotion regulation can occur before (anticipating), during (experiencing) or after (recalling) the generation of a positive emotion, the focus of our study is on the application of Gross’ extended model to the regulation of ongoing positive emotions suggested by Quoidbach et al. (thus, experiencing) (from line 89, from line 145).

*2. More justification should be provided about how and why the use of specific positive emotion regulation strategies should predict more positive emotions over time. Authors should explain what strategies they examine and why these may be useful to increase positive affect. They should mention that they only include ‘adaptive’ strategies given that maladaptive ones (e.g., dampening, suppressing, etc.) may curtail positive affect. Authors should incorporate other longitudinal studies that have shown that savoring types of ER strategies increase positive emotions over time. Examples include: Gentzler, Morey, Palmer, & Yi, 2012; Hurley & Kwon, 2013; Jose, Lim, Bryant, 2012; Langston, 1994.*

We completely agree with the reviewer’s point. The rationale behind the selection of the strategies was not adequately developed. In order to address this issue, two main changes were made.

First, and according to reviewer#1 suggestion, a more detailed explanation about the classification of positive strategies has been provided, which was based on the work by Quoidbach et al. (Quoidbach, Mikolajczak and Gross, 2015), who applied Gross’ emotion regulation model to the regulation of positive emotions (from line 192; from line 198).

- Cognitive change: In this work, the authors provide count blessing (“*Are you realizing how lucky you are or are you taking the situation for granted?*”) as an example of cognitive change in response to positive emotions, which is described as the attempt to “*change how one appraises the situation in order to alter its emotional significance”* by “*appraising the situation as a special moment*” and suggesting that “*positive emotions do not depend on situations per se as much as they depend on the way individuals interpret these situations*”. We therefore decided to include count blessing as a cognitive change strategy. Furthermore, we included broadening as a further example of cognitive change because it does reflect a way of appraising a positive situation as a special moment, which is consistent with the aforementioned definition. Moreover, broadening has been shown to be one of the most used strategies in response to positive emotions (Heiy and Cheavens, 2014).
- Attentional deployment: The authors define attentional deployment as the attempt to influence positive emotions by specifically directing our attention within a situation (“*What do you purposefully pay attention to during your date night*?”). Accordingly, we decided to include mindfulness (I’m trying to be focused on the present and to concentrate on how good I feel) and stimulus control (I’m trying to avoid all negative thoughts and stressors in order to focus and take the most of my positive emotions) as two strategies that reflects the attempt to modify one’s attentional focus in order to take the most out of a positive emotion.
- Response modulation: The authors define response modulation as the attempt to “*influence physiological, experiential, or behavioral responding as directly as possible*” and suggest social sharing and emotional expression as two examples of this category (“*Are you laughing and sharing your positive feelings with your partner or, conversely, hiding your emotions?”).* We therefore decided to include both strategies to represent this category.

Second, we have now included more information to justify the selection of the six strategies and to support their potential role on momentary affect (from line 198).

Besides, we have mentioned the lack of maladaptive strategies within the EMA assessment as a further limitation of the study (see response to comment 10) (from line 477). Finally, we have included the main findings of the studies suggested by the reviewer to further support the important emotional outcomes of positive emotion regulation (from line 95).

*3. Researchers should justify their sample size of 85 undergraduates and their chosen time frame for the study (3 measurements a day for two weeks). There is no rationale for either. It also isn’t clear if they eliminated anyone due to not completing a minimum number of ESM assessments.*

Thank you for raising these criticisms. Regarding the first issue, we decided the sample size by taking into account the sample sizes used in previous EMA studies on emotion regulation (see for example Pavani et al., 2015; Brans et al., 2013) (line 169). Regarding study design, we reviewed the past EMA literature to decide the best sampling frequency to adopt. However, no general rule was found, considering the incredibly huge variability among studies assessing ER through EMA protocols, ranging from once per day (see for example Nezlek et al. 2008) to ten times per day (see for example Kuppens et al. 2010). Because of the lack of shared guidelines, we decided to rely on the study by Heiy and colleagues (2015), which was used as a reference for the design of our study (especially for the ER items) and which for sure represents an important work within the field. Moreover, our previous studies adopting the same sampling frequency supports the feasibility of such design, which is reflected by the low burden and high compliance of participants (80.47%, Colombo et al., 2020a; 77.8%, Colombo et al., 2020b). Accordingly, more details about the selection of the sampling frequency have been included in the manuscript (from line 179).

Finally, we did not put a threshold on compliance levels in order to include or exclude participants. This information has been now included in the manuscript (line 182).

*4. Were the emotion scales labeled (the 1 and 5 endpoints or each number)?*

The endpoints of the Likert scales used to assess positive emotions were labelled (1=not at all; 5=a lot). We have now included this information in the manuscript (line 184).

*5. The ER questions need additional details so the reader can understand what was done and why. Examples should be provided. What is “stimulus control” as a ER strategy?*

We do completely agree that the selection of the strategies was not adequately detailed. As the reviewer will see, we have now clarified this issue. More specifically, and following reviewer#1 suggestion, we have now included an explanation of each ER category (attentional deployment, cognitive change, response modulation), which helped us introduce each of the positive strategy of our study. Furthermore, further references have been included in order to support their selection (from line 198).

*Why are difference scores computed and what do Time 0 to Time 1 represent? (A little more information is given in the results, but it should be defined in the method given it is first mentioned there.)*

In the revised manuscript, we have now included a clearer explanation about the change scores we calculated for each emotion regulation strategy (from line 229).

*There are existing measures of positive emotion regulation in the literature so better explaining the benefits of these items may be helpful*

Thank you for raising this point. As mentioned in the discussion, the use of single items to assess positive strategies might represent a criticism of the study. However, to the best of our knowledge, there aren’t validated items assessing state (e.g., momentary) positive regulation. The available questionnaires usually assess positive emotion regulation in a more general way (see for example the *Response Style to Positive Affect Scale*, which assesses emotion-focused or self-focused savoring; the *Savoring Belief Inventory*, which only explores savoring in terms of anticipating, experiencing or recalling positive states; or the *Emotion Regulation Profile-Revised*, which consists of narratives rather than items). Additionally, most of the available emotion regulation questionnaires relies on the idea that emotion regulation is a trait of a person, thus assessing emotion regulation as a cross-situational tendency. Only a few questionnaires have been developed to assess state emotion regulation (i.e., the momentary adoption of strategies in relation to a specific event, which would fit better with the use of EMA), but only in relation to negative emotions (see for example the *Brief State Rumination Inventory*, Marchetti et al., 2018; or the *State Difficulties in Emotion Regulation Scale*, Lavender et al., 2017). Finally, there is evidence showing that longer EMA questionnaires usually imply higher perceived burden and lower compliance to the experimental protocol, which further support our choice to use single items. Thus, integrating a full questionnaire of positive regulation in the EMA would have been counterproductive.

Broadly talking, the use of single items isn’t just a limitation of our study, but a very important issue of the EMA literature. Indeed, almost all EMA studies rely on the use of ad-hoc items, thus raising important issues regarding their construct validity. We acknowledge this criticism, and we recognise that there is the need to further expand this research field by creating validated measures to be used in momentary assessments. These efforts have been already made in other research fields. An example is the *Distress Thermometer*, a single measure which has been developed to assess “unpleasant experience of a mental, physical, social, or spiritual nature”. Similarly, validated items to assess emotion regulation should be created in the near future.

As the reviewer will see, we have tried to incorporate the aforementioned considerations in the manuscript and to better justify the use of single items (from line 484).

*6. Descriptives for the emotion and ER rates should be provided (maybe in Table 1).*

The descriptives for the emotions and ER rates have been included in Table 1, as suggested by the reviewer.

*7. In Table 2, the notations to flag significant associations are included but they don’t actually use them in the table. Instead they write out the p-values. However, given the table is very hard to read, using asterisks is recommended.*

Thank you for pointing out this mistake. We have now corrected the table by including the asterisks and deleting the p-values. We also modified the other tables, consistently.

*8. Researchers tested additional hypotheses not outlined in the introduction. These should not take the reader by surprise, so these should be mentioned and justified earlier.*

We completely agree with the reviewer. Indeed, the moderation of strategy category on the reciprocal influence between positive ER and PE was not mentioned among the hypotheses. We have now included it (from line 16\).

*9. The benefits of sharing positive experiences also depend on how the recipients respond to the news (Shelly Gable’s research). Therefore, recipients’ supportive v. less supportive responses may mitigate its positive effects sometimes.*

This is a very interesting point, which further explains the results observed in relation to the use of social sharing. We have now included it in the discussion of the results (from line 448).

*10. Additional limitations include not including maladaptive responses to PA (e.g., dampening) and negative affect. When people are lower on PA, do they also do more ineffective ER? It seems likely that they do not solely use the types of ER strategies that happened to be assessed here.*

Thank you for the suggestion. We have now underlined the lack of items to assess maladaptive strategies and negative affect as two further limitations of the present study. Regarding the latter, one of the reviewers suggested that, the lack of measures about negative affect, does not allow to conclude that “upregulating PE may serve as a mechanism to repair mood and reduce the experience of unpleasant affective states”. Indeed, the fact that we only assessed momentary PE levels does not allow to fully comprehend the reciprocal influence between positive emotion regulation and momentary affect (both positive and negative). We therefore decided to move this statement to the limitations of the study. More specifically, we state that, despite the results might support the mood-repair theory, such conclusion can’t be confirmed because of the lack of information regarding momentary negative affect (from line 470).

*11. There are many grammatical issues with this paper, so these would need to be corrected.*

The manuscript has been edited by an English-speaking native in order to correct grammatical issues.
