# Supplementary material for: Savoring the present: The reciprocal influence between positive emotions and positive emotion regulation in everyday life
Source: PLoS One. 2021 May 11;16(5):e0251561. doi: 10.1371/journal.pone.0251561 (PMC8112694; doi:10.1371/journal.pone.0251561)
Supplement: Attachment — Submitted filename: Response to reviewers_rev2.docx [file pone.0251561.s002.docx]

**Reviewer #1**

*The authors were somewhat responsive to earlier concerns. However, certain points were not addressed as expected or the changes resulted in new problems. Although I believe this paper has several strengths and could contribute to the literature, the remaining issues dampen my enthusiasm but could be addressed in the future.*

We are very pleased to know that the reviewer believes that the manuscript might represent an important contribution. Also, we are very grateful for the insightful points outlined and we have enthusiastically tried to address all concerns. In our resubmitted manuscript, we have highlighted areas of substantive changes in yellow, so that the editor and the reviewer can easily identify them. Material that has been moved around from one place to another is in **bolded green font**.

*Terms are not defined in the introduction when first mentioned – capitalizing, savoring, AD, CC, RM. It doesn’t make sense to wait until the method to cite literature explaining what the strategies are and what they relate to.*

Thank you very much for the suggestion. We have now included in the manuscript the definitions of the terms mentioned by the reviewer (see for example line 66 or line 100). Furthermore, we have moved the literature supporting the six positive strategies from the methods to the introduction (from line 164). We do agree that it is more consistent to provide this information to the reader when introducing our study.

*Relatedly they do not justify the particular emotion regulation strategies that they focus on. Some of this text (justifying why these strategies would relate to more PE) is included in the discussion, but it would be helpful to have this information in the introduction and state they’re excluding maladaptive ER.*

We have now moved to the introduction the paragraphs justifying the selection of the six strategies and discussing their association with PE (“The current study”) (from line 164), and we have included a more detailed explanation about the rational used to select categories and strategies. Categories were selected based on the review by Quoidbach et al. (2015), who points out that “*Regarding short-term increases in positive emotions, our review found that attentional deployment, cognitive change, and response modulation strategies have received the most empirical support, whereas more work is needed to establish the effectiveness of situation selection and situation modification strategies”* (from line 153)*.* Strategies were selected in order to explore these 3 categories of emotion regulation (two strategies for each category) and according to the literature supporting the association between these specific strategies and PE. Furthermore, and as suggested by the reviewer, in the introduction we have now underlined that maladaptive ER has not been investigated (lines 151-152). Besides, we also explain that, although people’s repertoire to deal with positive emotions is quite large, we only focused on a limited number of strategies in order to reduce participants’ burden and efforts to complete 3 daily EMA assessments (from line 147).

Finally, it is true that in the discussion we mention three further studies about the influence of positive regulation (more specifically, response modulation) on PE (58, 59, 60). However, these studies are very specific, and they are presented in the discussion in order to explain posteriori why response modulation strategies had a lesser influence on PE in our study.

*This sentence needs more explanation given this study doesn’t study mindfulness, self-esteem, and autonomy: “ Furthermore, intense use of mindfulness has been shown to predict higher levels of daily autonomy (24), whereas positive reappraisal has been associated with greater self-esteem and well-being (25).” Although there is a mindfulness type of item as ER, readers don’t know that until the method, so the sentence appears mostly unrelated.*

After stating that “*The implementation of positive emotion regulation has been shown to be beneficial for mental health*”, our aim was to provide some examples of positive strategies that have been found to be associated with higher well-being, such as counting blessings, capitalizing, mindfulness and positive reappraisal. As the reviewer will see, we have rephrased the paragraph in order to make our objective clearer for the reader (from line 93). We have also deleted the specific terms “self-esteem” and “autonomy”, as these constructs are not explored in our study and might be misleading for the reader.

*A major problem in the paper remains unclear description and treatment of ER strategies. If the individual strategies are being collapsed into categories, why isn’t this done consistently? Some analyses examine ER as individual strategies (all 6) and others include combined strategies (AD, CC, RM) but there is no rationale for the switch back and forth.*

We agree with the reviewer that this point was not enough clear in the manuscript. As explained in the previous comment, the rational used in this study was mainly based on the results of the review by Quoidbach et al., (2015), who found attentional deployment, cognitive change and response modulation to be more effective in increasing PE in the brief term. Starting from these 3 categories, we selected two strategies for each category based on the previous literature relating positive emotion regulation to positive emotions. This rational allowed us to explore the association between positive emotion regulation and positive emotions both at a strategy and category levels: In other words, not only the association between the use of 6 positive strategies and PE, but also the influence of strategy category on the experience positive emotional states (i.e., the intrinsic attentional, cognitive or behavioural nature of the strategies). We have now modified the manuscript in order to clarify this rational. As the reviewer will see, we have included a more detailed explanation of the strategies/categories in order to explore the association between positive emotion regulation and PE both at a strategy and category levels (from line 153), as well as a better elaboration of the 3 aims of the study (from line 197). Furthermore, the statistical analyses section has been improved in order to explicitly match each statistical step to the three aims of the study (lines 294-295; lines 309-310; lines 320-321).

*The use of acronyms for AD, CC, and RM are confusing as they are not well known. Can these be written out?*

We have now substituted acronyms with the entire name of each strategy category.

*It is not clear from Table 1 that authors are correlating scores that define other scores (e.g., expressing and sharing are RM, so that’s why the correlations are .90). This should be explained in a note or authors should pick one (either the aggregate or individual scores) and use one throughout the paper.*

Following the reviewer’s suggestion, we have improved the caption of Table 1 in order to include a note about how emotion regulation categories were calculated. We have also modified the table in order to emphasize and separate the two levels (strategy and categories). Furthermore, we have moved the EMA items from the methods to Table 1, in order to make the manuscript easier to read and reduce the use of parentheses, as suggested by the reviewer in the comment below.

*Do authors test for significant differences between strategy use? It is not clear based on how it is written and it’s not clear how much value this text adds.*

In the previous version of this manuscript, we did not test for significant differences between the mean use of our strategies of interest. The paragraph pointed by the reviewer was included only to display descriptive statistics. We added this paragraph to be consistent with previous studies on emotion regulation in everyday life, that contained such a paragraph. Nevertheless, as the reviewer suggested, this paragraph does not add enough value to our manuscript to be conserved. More specifically, this paragraph described statistics that are already easily accessible on Table 1. For this reason, rather than testing mean differences which is not very informative and valuable in relation to the aims of our study, we have decided to remove the paragraph.

*Why is T1 PE controlled for when predicting ER strategies from PE T0? Authors should have a good rationale for including it and explain it in the paper (or instead not include it as a covariate).*

The rational for including PE at t1 as a covariate was already described in the “statistical analyses” section. We have rephrased the sentence in order to make it clearer (lines 302-308). Not controlling for PE t1 could produce a biased estimation of the effect of PE t0 on subsequent strategies. As PE t1 was related to (1) PE t0 and (2) strategy changes from t0 to t1, it could represent a confounding variable when attempting to determine the specific relationship between PE t0 and strategy changes from t0 to t1. Therefore, to ensure that the effect of PE t0 on strategy changes from t0 to t1 was not actually explained by PE t1’s relationships with both variables, we controlled for PE t1.

*Conversely, it seems that T0 PE should be included as a covariate when predicting PE T1 from ER. Was that done? Where all 8 strategies run in separate models?*

PE at T0 was included as a covariate when predicting PE at T1 from ER, and the rational for including it was discussed in the “statistical analyses” section. We have underlined this part in the manuscript (lines 312: “PE at t0 was also included as a control variable to neutralize the so-called regression towards the mean effect”). In this case, we only ran one model containing change in the use of each strategy from t0 to t1 as independent variables to predict PE at T1. This information has been clarified in the manuscript (line 310). As the manuscript included a table to report the results of all the analyses performed, we have also included a table to report these results (table 3), which in the previous version of the manuscript were only reported in the text.

*Why isn’t AD included in Tables 3 and 4 and corresponding analyses? More information should be included about the models testing these hypotheses, and potentially including analyses in a table given they are major findings of the paper.*

The analyses reported in table 4 and table 5 explored the presence of a significant categorical (strategy category: attentional deployment, cognitive change, response modulation) by continuous (PE at t0 for table 4 and change in strategy intensity at t1 for table 5) interaction. A significant interaction means that the slope of the continuous variable is different for one or more levels of the categorical variable. Results are always to be interpreted in relation to the reference group, which in our case was attentional deployment (and that’s why attentional deployment is not reported). We have now modified the tables and included a note in the caption in order to clarify this point (from line 398).

*The rationale for the analysis in Table 4 is not provided and the analyses and results are not clear. It is recommended these are removed or set up as a hypotheses and clarified. The authors said they added the analyses in Table 3 and 4 as hypotheses, but the one sentence added at the end of the introduction is not clear in terms of corresponding to Table 4.*

Thank you for this suggestion. We have now included more details to describe the third objective of the study (that is, whether strategy category moderates the association between PE and positive emotion regulation) and we have explicitly mentioned that the moderating role of strategy category will be explored in both direction (lines 210-217): Strategy category as a moderator of the association between PE at t0 and positive regulation at t1 (which refers to the analyses reported in table 4); and strategy category as a moderator of the association between positive regulation at t0 and PE at t (which refers to the analyses reported in table 5). Also, we have modified the statistical analyses section in order to explicitly match the analyses conducted to explore the third aim of the study (from line 320).

*The description of some of the results is not summarized as clearly as it could. For example: “Interestingly, the tendency to adopt positive strategies to increase PE was less effective when using RM strategies (e.g., sharing and emotional expression).” Was this difference significant? What was it less effective than?*

The difference was statistically significant, as reported in the results section We have now revised this sentence in order to clarify the meaning and interpretation of this result (from line 471).

*In general, it is hard to see breaking of paragraphs, but it appears the last paragraph of the paper goes on for two pages. They should break it into two paragraphs (last 3 sentences could be on their own).*

The whole manuscript has been revised in order to make paragraphs more concise.

*Several typos are present. For example: a. Langstone instead of Langston; b. In the measures paragraphs there are multiple typos, and use of so many parentheses is very hard to follow.*

Thank you for pointing out the typos, we have now corrected them. Furthermore, the manuscript has been edited once more by an English-speaking native in order to correct grammatical issues.
